# Smad7 and Colorectal Carcinogenesis: A Double-Edged Sword

**DOI:** 10.3390/cancers11050612

**Published:** 2019-05-01

**Authors:** Edoardo Troncone, Giovanni Monteleone

**Affiliations:** Department of Systems Medicine, University of Rome “Tor Vergata”, 00133 Rome, Italy; troncone.edoardo@gmail.com

**Keywords:** TGF-β1 signaling, antisense oligonucleotides, colorectal cancer, colon cancer, colitis-associated cancer

## Abstract

Colorectal carcinogenesis is a complex process in which many immune and non-immune cells and a huge number of mediators are involved. Among these latter factors, Smad7, an inhibitor of the transforming growth factor (TGF)-β1 signaling that has been involved in the amplification of the inflammatory process sustaining chronic intestinal inflammation, is supposed to make a valid contribution to the growth and survival of colorectal cancer (CRC) cells. Smad7 is over-expressed by tumoral cells in both sporadic CRC and colitis-associated CRC, where it sustains neoplastic processes through activation of either TGFβ-dependent or non-dependent pathways. Consistently, genome-wide association studies have identified single nucleotide polymorphisms of the Smad7 gene associated with CRC and shown that either amplification or deletion of the Smad7 gene associates with a poor prognosis or better outcome, respectively. On the other hand, there is evidence that over-expression of Smad7 in immune cells infiltrating the inflamed gut of patients with inflammatory bowel disease can elicit anti-tumor responses, with the down-stream effect of attenuating CRC cell growth. Taken together, these observations suggest a double role of Smad7 in colorectal carcinogenesis, which probably depends on the cell subset and the biological context analyzed. In this review, we summarize the available evidences about the role of Smad7 in both sporadic and colitis-associated CRC.

## 1. Introduction

Colorectal cancer (CRC) is one of the most common forms of cancer and the second cancer-related mortality cause worldwide [1]. More than two thirds of CRCs develop in absence of clear genetic risk factors (sporadic CRC) while in nearly 2% of cases CRC develops in patients with ulcerative colitis (UC) or Crohn’s disease (CD) (colitis-associated cancer, CAC), the major inflammatory bowel diseases (IBD) in human beings. The remaining forms of CRC have a clear genetic background. About 5% of CRCs are marked by genetic mutations, which outline a hereditary syndrome. Examples are the Lynch syndrome, which is characterized by a germline mutation in one of the DNA mismatch repair genes, and the Familiar Adenomatous Polyposis, in which patients carry a mutated adenomatous polyposis coli (APC) gene. Nearly one fourth of CRC patients manifest an increased family risk without a known genetic syndrome [2,3]. Several environmental factors have been investigated as risk factors for CRC, and most of them are part of the so-called “western” life-style, which for decades has characterized the habits of persons living in high-income countries. For instance, consumption of red and processed meats and alcohol, and obesity show the stronger association with CRC, while physical activity and consumption of fiber are protective [1,4]. Consistently, in the last decades, countries undergoing major development transition, such as Baltic countries, China, Russia and Brazil, have documented an increase of both CRC incidence and mortality [5]. 

Although the exact sequence of molecular events that triggers and sustains colon carcinogenesis remains to be ascertained, there is evidence that multiple genetic mutations are needed to proceed from a normal colon epithelium to adenoma and then adenocarcinoma [6]. Mutations in the APC gene, which characterizes both inherited and sporadic CRC, occur early in the carcinogenetic process, while p53 mutations generally occur later [7]. During the neoplastic process, colonic epithelial cells progressively lose physiological control of proliferation, programmed death and polarization, acquiring the phenotype of invasive cancer cells [6,8].

The increasing availability of screening programs is improving the diagnostic rate of pre-neoplastic lesions and early stage CRC, thus providing the possibility of an early and effective therapy. Nonetheless, the management of advanced disease is still a difficult challenge for clinicians. In recent years, a more in-depth knowledge of molecular mechanisms underlying the malignant transformation and cancer progression led also to the identification of molecules and mediators, which can be targeted for therapeutic interventions, thus advancing the way to manage CRC patients. A clear demonstration of this concept comes from the use in clinical practice of monoclonal antibodies targeting the Epithelial Growth Factor (EGF) receptor (cetuximab, pananitumumab) in patients bearing a non-mutated form of KRAS, or the Vascular Epithelial Growth Factor (VEGF) (bevacizumab) [9,10,11]. Indeed, targeting specific molecules in selected and profiled patients leads to a more personalized therapeutic approach, which helps maximize benefits and minimize adverse effects. In this review, we summarize the available data on the dual role of Smad7, also known as mothers against decapentaplegic homolog 7 (MADH7), in colonic carcinogenesis, and discuss potential advantages and drawbacks in targeting such a molecule in CRC patients. 

## 2. TGF-β1/Smad7 Axis in Intestinal Mucosa

Smad7 is an intracellular protein, which has been traditionally considered as a negative regulator of Transforming Growth Factor (TGF)-β1 [12]. TGF-β1 biological functions are mediated by two transmembrane receptors, namely the TGF-β1 Type 1 receptor (TβR1) and TGF-β1 Type 2 receptor (TβR2) [13]. TGF-β1 binds to TβR2 and triggers auto-phosphorylation of the receptor, thus promoting the recruitment of TβR1 and formation of a heterodimer. Subsequently, the glycine/serine kinase activity of TβR2 determines the phosphorylation of the regulatory domain of TβR1 with the downstream effect of activating the TβR1-TβR2 complex and triggering phosphorylation of two intracellular proteins, named Smad2 and Smad3. Phosphorylated Smad2/Smad3 form heterodimers with Smad4 and move to the nucleus, where such a complex controls the transcription of several target genes [13,14]. Smad7 interferes with TGF-β1 signaling in several ways. First, Smad7 can prevent the phosphorylation of Smad2/3 by binding to TβR1 and competing with Smad2/3 for the catalytic site of phosphorylation [15,16]. Smad7 can recruit phosphatases to TβR1 and cause inactivation of the site through de-phosphorylation [17]. Moreover, Smad7 can interact with E3 ubiquitin ligases SMAD ubiquitination regulatory factor (SMURF)-1/2 and facilitate TβR1 degradation in an ubiquitination-driven proteasome-mediated manner [18,19]. In the nucleus, Smad7 can directly bind the promoter of TGF-β1 target genes and disrupt the TGF-β1-mediated interaction between Smad complex and DNA [20]. 

TGF-β1 is constitutively produced in the human gut, where it exerts a plethora of biological functions by targeting both immune and non-immune cells. For instance, TGF-β1 controls enterocyte proliferation and margination and stem cell division, and stimulates stromal cells to produce fibrogenic mediators and regulators of extracellular matrix (ECM) deposition. Indeed, TGF-β1 promotes differentiation of mesenchymal cells in myofibroblasts, which display contractile activity and produce collagen and fibronectin, thereby facilitating wound repair [21,22]. Therefore, TGF-β1 is considered as a major pro-fibrogenic cytokine and a poorly controlled TGF-β1 activity has been involved in development of intestinal fibrosis and strictures, which may complicate the natural history of CD [23,24,25]. TGF-β1 regulates the function of several immune cells, both in the adaptive and innate compartment. TGF-β1 inhibits the function of antigen presenting cells and effector T cells, helping to keep immune intestinal homeostasis [12,26,27,28,29,30]. Mice bearing a specific deletion of TβR2 in T cells or over-expressing a dominant-negative form of TβR2 are unable to respond to TGF-β1 and exhibit a phenotype characterized by systemic autoimmunity and severe colitis [31]. TGF-β1 inhibits Th1 differentiation by directly downregulating the transcription factor Tbet, a master regulator of T helper (Th) 1 cell polarization [28]. Moreover, TGF-β1 downregulates the expression of interleukin (IL)-12 receptor β2, thus preventing the expansion of Th1 cell responses driven by IL-12, a crucial cytokine for the induction of Th1-type immunity [32]. In a similar manner, TGF-β1 downregulates GATA3, a master regulator of Th2 cell differentiation and stimulates T cell differentiation towards a regulatory phenotype, thus favoring the generation of the so-called regulatory T cells (Tregs), which exert immune suppressive functions [26,27,33]. In particular, low levels of TGF-β1 promote the expression of the transcription factor forkhead box P3 (Foxp3), a master regulator of Tregs, thus polarizing CD4+ T lymphocytes towards a regulatory phenotype [29,30]. In this context, the inhibitory activity of Smad7 on TGF-β1 signaling impairs Tregs differentiation, and post-transcriptional modifications of Smad7, such as cylindromatosis (CYLD)-mediated deubiquitination, can dramatically influence the effects of TGF-β1 on Tregs development [34]. In addition to the aforementioned effects, TGF-β1 contributes to the differentiation of Th17, controls memory CD8+ T cells, regulates B cells and plasma cells functions and is involved in the regulation of several immune cells of the innate compartment, such as intestinal dendritic cells, macrophages and innate lymphoid cells [12]. All these TGF-β1-mediated functions are crucial for the balancing of the immune response against pathogens, as well as for the regulation of the anti-tumor activity of immune cells. For a detailed description of the functions of TGF-β1 in the gut, the reader is directed towards recent reviews [12,35]. 

Given the central role played by TGF-β1 in the induction and maintenance of intestinal homeostasis, many researchers have investigated the expression and activity of such a cytokine in IBD and CRC. Initial studies characterized the RNA expression of the cytokine in IBD and showed that TGF-β1 transcripts were over-expressed in inflamed mucosa of UC patients and CD patients as compared to uninflamed colonic mucosa [36]. Nonetheless, subsequent studies documented a defective TGF-β1 activity in IBD, which was associated with high Smad7. Similar defects were seen in mice with IBD-like colitis. The functional relevance of such data was supported by the demonstration that knockdown of Smad7 with an antisense (AS) oligonucleotide re-established TGF-β1 activity and suppressed inflammatory signals both in vitro and in vivo [37,38,39,40]. Overall, these findings suggest that, in the gut, high Smad7 contributes to block TGF-β1-mediated immune suppression with the downstream effect of amplifying signals that sustain mucosal inflammation. 

Many studies have highlighted the ambivalent role of TGF-β1 in CRC. TGF-β1 can behave as a strong tumor-suppressor by inhibiting epithelial cell growth and maintaining their differentiation state in the initial stages of colonic carcinogenesis [41]. However, the anti-tumor effect of TGF-β1 is often bypassed by cancer cells, which may display a wide range of mutations in key points of the TGF-β1 signaling pathway, such as those regarding TRβ2 or Smad2/Smad4 genes [42,43,44]. In contrast, in the advanced stages of the disease, TGF-β1 can promote epithelial-to-mesenchymal transition (EMT), a process by which epithelial cells lose cell-cell adhesion, apico-basal polarity and can acquire motility and chemotherapy resistance [45,46,47]. In this context, it has been demonstrated that TGF-β1 directly controls several transcription factors involved in the EMT process, such as zinc finger E-box-binding homeobox (ZEB) 1 and ZEB2, Snail, Slug, and Twist, all of which regulate the expression of epithelial junction proteins E-cadherin and occludins [48,49]. TGF-β1-mediated downregulation of epithelial junction proteins leads to a profound reorganization of the actin cytoskeleton and promotes the acquisition of a mesenchymal phenotype with a front-rear polarity and motility. TGF-β1 can also stimulate neoplastic cells to produce metalloproteases and ECM components, which contribute to the invasive properties of neoplastic cells and to the reconstitution of tumor microenvironment after invasion [41]. At this stage of carcinogenesis, the pro-tumorigenic activity of TGF-β1 could be increased by changes in the inhibitory activity of Smad7 [50]. Katsuno and colleagues have recently shown that the methyltransferase protein arginine methyltransferase (PRMT) 1 promotes Smad7 methylation, thereby decreasing binding of Smad7 to TβR1 and facilitating TGF-β1 signaling and TGF-β1-mediated EMT [50]. Moreover, TGF-β1 may stimulate tumorigenesis by inducing angiogenesis, mainly through activation of activin receptor-like kinase (ALK) 1 and endoglin, [35]. Finally, as mentioned above, TGF-β1 restrains both adaptive and innate immune responses and promotes the differentiation of immune-suppressive Tregs. Such effects on the immune system can dampen anti-tumor immunity and finally sustain tumorigenesis [35]. However, the role of Foxp3-expressing Tregs in CRC is controversial, with some data suggesting a protective effect on tumorigenesis [51,52,53]. In this context, Saito and colleagues demonstrated that tumor infiltrating lymphocytes (TILs) expressing high levels of Foxp3 showed suppressive functions and were able to dampen the anti-tumor immune response, while TILs expressing low levels of Foxp3 secreted pro-inflammatory cytokines and did not show suppressive properties. Consistently, CRC infiltrated by high FoxP3-TILs showed worst prognosis compared to tumors infiltrated by low FoxP3-TILs [54].

## 3. Smad7 and Colitis-Associated Colon Cancer

CRC can complicate the natural history of patients with colonic IBD (colitis-associated cancer, CAC), with a cumulative risk that is related to disease extension, duration and severity of inflammation [3,55]. Early population-based studies and hospital cohorts reported an overall CRC risk in UC patients of 3.7% and a standard incidence ratio (SIR) of 5.7% [56,57]. More recent studies have substantially lowered the magnitude of the risk, with some reports documenting a risk comparable to the general population [58]. This has been linked to various factors, including a better control of the chronic mucosal inflammation with innovative and powerful therapeutic approaches (e.g., biologics), the adoption of surveillance programs in high-risk patients and timely colectomy.

It is widely known that, even in the gut, inflammation is strongly linked to carcinogenesis, with antithetical effects depending on the characteristics of inflammation (i.e., cytokine pattern and immune cells involved) and the stage of the carcinogenesis process. Chronic inflammation enriches the colonic mucosa of reactive nitrogen species (RNS) and reactive oxygen species (ROS), which can lead to DNA damage, cell transformation and initiation of cancer [59]. Moreover, during inflammation, many cytokines secreted by immune and non-immune cells infiltrating the inflamed tissue can stimulate epithelial cell proliferation, inhibit cell death and facilitate tumor progression. It is now recognized that the pro-tumorigenic effect of most cytokines, which directly targets neoplastic cells, is mediated by activation of nuclear factor (NF)-kB and signal transducer and activator of transcription (STAT) 3 [60,61]. For instance, Greten et al. showed that inhibitor of kB kinase (IKK)-β-mediated NF-kB activation in myeloid cells stimulates the expression of cytokines and growth factors, which promote CAC induction and progression. Consistently, IKKβ deletion in myeloid cells or in epithelial cells in CAC murine models associates with reduced tumor size or reduced tumor numbers respectively, emphasizing the role of NF-kB in controlling epithelial cell apoptosis and in promoting pro-tumorigenic inflammatory signals [61]. Further work focusing on the role of specific mediators involved in CAC progression has contributed to show that IL-6, mainly produced by intestinal dendritic cells and macrophages, is crucial for tumor growth and production of pro-tumorigenic factors. Indeed, IL-6 inhibition or deletion in transgenic CAC murine models reduces STAT3 activation thus resulting in reduced cancer growth [60]. Several studies have also emphasized the role of IL-17A in colon carcinogenesis. IL-17A is produced by several immune cell sub-types, including Th17 cells [62]. IL-17A directly promotes the progression of early dysplastic lesions into adenomas and adenocarcinomas, and elevated expression of such cytokine, together with IL-23R, correlates with the worst prognosis and progression to metastatic disease in early stages of human CRC [63,64]. On the other hand, the immune system is able to recognize tumor antigens and mount vigorous immune responses against tumor cells eliminating them, through the so-called “immune-surveillance” [65,66,67]. This concept has been successfully translated in the development of immune checkpoint inhibitors that trigger tumor rejection by activating cytotoxic T lymphocytes (CTLs). Such drugs are currently used for several types of solid cancer (e.g., melanoma, non-small cell lung cancer, kidney and bladder cancer) [66].

Since, in IBD mucosa, Smad7 is over-expressed in effector T cells and positively regulates production of T cell-derived cytokines/molecules, which could potentially influence tumor cell growth and survival, we investigated the role of Smad7-expressing immune cells in the control of CAC development. Initially, we showed that Smad7-expressing CD4+ T lymphocytes were more abundant in the colonic mucosa of IBD patients with CAC as compared to normal controls, even though their number was reduced as compared to that seen in IBD patients with uncomplicated disease [68]. Next, we developed transgenic mice over-expressing Smad7 in T cells and assessed the susceptibility of such animals to develop colitis following oral administration of dextran sodium sulfate (DSS) and DSS-driven CAC. Smad7 transgenic mice showed more severe intestinal inflammation with an intense mucosal infiltrate of cytokine-secreting T cells and high levels of interferon (IFN)-γ, IL-6, and IL-17A as compared to control mice following DSS administration but, surprisingly, were less susceptible than wild-type to the development of CAC. Interestingly, tumors of Smad7 transgenic mice showed higher levels of IFN-γ as compared to wild-type mice, while the other cytokines analyzed were downregulated, indicating a prevalent IFN-γ-associated immune response within the tumor environment of transgenic mice. Consistently, genetic deletion of IFN-γ reversed the protective effect of Smad7 on carcinogenesis, thereby confirming a central role for this cytokine in determining the anti-tumorigenic effect of Smad7 over-expressing T lymphocytes [68]. These data were then confirmed in a different model of carcinogenesis, in which Smad7 transgenic mice subcutaneously injected with syngenic MC38 colon carcinoma cells developed less tumors as compared to wild-type littermates, with this protection being dependent on CD4+ T cells [69]. Smad7 over-expression in T cells promotes infiltration of the tumor environment by Tbet/RAR-related orphan receptor (ROR)-γt double-positive CD4 T cells, which produce high levels of tumor necrosis factor (TNF)-α and IFN-γ, but lower levels of IL-17A. Since IL-17A is able to inhibit CRC cell apoptosis both in vitro and in vivo, we demonstrated that the reduced IL-17A levels allow effective TNF-α-mediated killing of cancer cells. Although the exact mechanism by which Smad7-expressing T cells negatively regulate CAC remains to be ascertained, it is plausible that the robust T cell response developing in the presence of Smad7 can facilitate the activation of signals that eventually kill epithelial cells. This hypothesis is supported by the demonstration that Smad7-transgenic mice exhibited accumulation of cytotoxic CD8+ T cells and Natural Killer T (NKT+) cells in the mucosa. More recently, Pallangyo and colleagues documented a tumor-suppressive function of IKKβ/NF-kB in cancer-associated fibroblasts (CAF) in murine models of CAC and sporadic cancer [70]. Fibroblast-restricted deletion of Ikkβ stimulated intestinal epithelial cell proliferation, suppressed tumor cell death, and induced angiogenesis, ultimately resulting in accelerated tumor growth, through a mechanism mediated by the hepatocyte growth factor (HGF). Notably, overexpression of Smad7 in Ikkβ-deficient fibroblasts prevented HGF secretion thus confirming the tumor-suppressor role of Smad7 expressed by non-epithelial cells [70]. 

## 4. Smad7 and Sporadic Colorectal Cancer

A consistent body of evidence has linked Smad7 to several types of sporadic cancers. For example, Smad7 is highly expressed in esophageal, gastric and endometrial cancer, and generally associates with higher recurrence rate and poor prognosis [71,72,73,74]. A more pronounced expression of Smad7 has been documented in squamous cell carcinoma and human papilloma as compared to normal epidermis [75]. An analysis of 150 invasive breast cancer specimens showed that Smad7 expression positively correlated with tumor stage and size, and associated with an aggressive phenotype [76]. On the other hand, some experimental evidences indicate the Smad7 could act as a negative regulator of breast carcinogenesis [77,78,79]. More recently, data from 205 skin melanoma primary tumors showed that Smad7 was expressed in almost all the specimens, with a predominantly nuclear pattern, and high Smad7 expression was positively associated with several features of tumor aggressiveness (i.e., presence of ulceration, higher tumor thickness, higher mitotic rate) and independently predicted an unfavorable prognosis [80]. In pancreatic cancer, elevated expression of Smad7 associates with a better prognosis as compared to patients with lower expression, who show higher incidence of lymph node and liver metastasis after surgery [81]. However, some in vivo and in vitro studies support the hypothesis that Smad7 exerts a pro-tumorigenic effect in pancreatic cancer cells [82,83,84]. Indeed, transgenic mice with selective pancreatic Smad7 over-expression develop pre-malignant pancreatic ductal lesions similar to pancreatic intraepithelial neoplasia (PanIN) in early stages of life [85]. Taken together, these data highlight a dual role of Smad7 in different types of cancer with anti-tumorigenic or pro-tumorigenic effects according to the cancer biology and site of development.

Several studies have also analyzed the role of Smad7 in sporadic CRC. By gene dosage using real-time quantitative, Boulay and colleagues documented Smad7 deletion in 43% (77/178) of CRC biopsy samples and showed that patients with tumors in which deletions of Smad7 had been documented, had a low hazard ratio for death and relapse [86], clearly defining Smad7 as a negative prognostic marker in patients with CRC. Phipps and colleagues examined the relationship between 16 germline single-nucleotide polymorphisms (SNPs) associated with CRC incidence, and survival rate of 2611 CRC patients. It was shown that a SNP in the Smad7 gene (rs4939827), formerly associated with lower risk of incident CRC [87], was associated with reduced overall survival and disease-specific survival [88]. Similarly, rs4939827 was associated with decreased risk of incident CRC but worst prognosis with poorer survival after CRC diagnosis, thus reflecting the dual role of the TGF-β pathway on CRC initiation and progression. A large meta-analysis of 63 studies involving 187,181 subjects showed the association between Smad7 polymorphisms rs4939827, rs4464148 and rs12953717 and the increased risk of CRC [89]. Recently, a work from Hu and colleagues focused on susceptibility loci that could predict rectal cancer prognosis after surgery [90]. The analysis included data from 243 rectal cancer patients and confirmed that the SNPs rs12953717 and rs4464148, located in an intron of SMAD7 on 18q21, were significantly associated with rectal cancer recurrence. Moreover, patients with lower SMAD7 expression showed a longer disease-free survival [90].

There is evidence that the pro-tumorigenic effects of Smad7 in CRC are, at least in part, dependent on the inhibition of the tumor-suppressive properties of TGF-β1 in the normal epithelium. For instance, over-expression of Smad7 in the CRC cell line FET enhanced anchorage-independent cell growth and increased resistance against apoptosis through pathways dependent on TGF-β1 suppression. In particular, Smad7 interferes with the cell cycle by preventing TGF-β1-induced G1 arrest, inhibits downregulation of c-Myc, cyclin-dependent kinase (CDK) 4, and Cyclin D1, and blocks the expression of p21 (Cip1). Finally, Smad7 inhibits TGF-β1-mediated downregulation of retinoblastoma protein (Rb) phosphorylation. Moreover, immunocompromised mice injected subcutaneously with Smad7-overexpressing FET developed visible tumors compared with mice injected with normal FET, demonstrating increased tumorigenicity of the transgenic clones [91]. A work from the same group showed that Smad7 over-expressing FET also exhibited increased metastatic potential, as immunocompromised mice injected in the spleen with such a modified cell line were more prone to develop liver metastasis [92]. Moreover, genetically modified FET cells that metastasized to the liver showed an altered expression pattern and localization of several junction proteins, such as claudins, E-cadherin and ZO-1. Li et al. confirmed the pro-tumorigenic effect of Smad7 in CRC lines and identified microRNA-25 (MiR-25) as a potential inhibitor of Smad7 [93]. MiR-25 is dysregulated in several human cancers, and it is downregulated in human CRC as compared to normal, non-tumor tissue. In their work, Li and colleagues identified Smad7 as a target of MiR-25 through interaction with Smad7 3’-UTR, and showed that over-expression of the miRNA resulted in a marked decrease of Smad7 levels. Moreover, MiR-25-mediated Smad7 downregulation decreased tumorigenicity of HCT-116 CRC cells in a xenograft model [93]. We have previously shown that Smad7 is up-regulated in several human CRC cell lines as well as in human sporadic CRC tissue as compared to non-tumor tissue [94]. By the staining of sporadic CRC sections, we also showed that cancer cells are the major producers of Smad7 within the tumor microenvironment. Knockdown of Smad7 with AS oligonucleotide in DLD-1 and HCT-116 CRC cell lines led to a progressive accumulation of cells in the S phase of the cell cycle and decrease of cells in G0/G1, which ultimately resulted in enhanced cell death. Moreover, inhibition of Smad7 reduced the in vivo growth of HCT-116 cell-derived xenografts. Altogether these findings indicate that DNA replication of CRC cells is inhibited following Smad7 knockdown and support the hypothesis that Smad7 plays a positive role in CRC proliferation.

Interestingly, CRC cell lines used in such experiments are unresponsive to TGF-β1, and stimulation in vitro with TGF-β1 or anti-TGF-β1 did not impair the anti-proliferative effect of Smad7 AS in CRC cells, raising the possibility that Smad7 regulates CRC cell behavior through a TGF-β1-independent mechanism [94]. A more in-depth analysis of the mechanisms underlying the proliferative effects of Smad7 on CRC cells showed that Smad7 knockdown enhanced phosphorylation of eukaryotic translation initiation factor 2 α (eIF2α), a transcription factor responsible for cell cycle regulation [95]. Phosphorylation of eIF2α determines in turn the increase of expression of activating transcription factor 4 (ATF4) and CCAAT/enhancer binding protein homology protein (CHOP). In vitro experiments showed that knockdown of the serine-threonine protein kinase RNA abrogated Smad7 AS-induced eIF2α phosphorylation and ATF4/CHOP induction, and consequently prevented CRC cell death [95]. A recent work from Wang and co-workers reported some different results and showed that Nuclear reporter subfamily 2, group F, and member 2 (NR2F2), a pleiotropic mediator involved in several physiological processes and up-regulated in CRC cells, induced a TGFβ1-dependent EMT through Smad7 inhibition. Indeed, luciferase assay and chromatin immunoprecipitation showed that NR2F2 directly promotes the expression of the miRNA miR-21, which in turn inhibits Smad7 expression. In the model proposed, the NR2F2/miR-21-mediated Smad7 inhibition finally lead to TGFβ1-dependent EMT, thereby enhancing migration and invasion of CRC cells and promoting metastasizing process [96].

**Figure 1:**
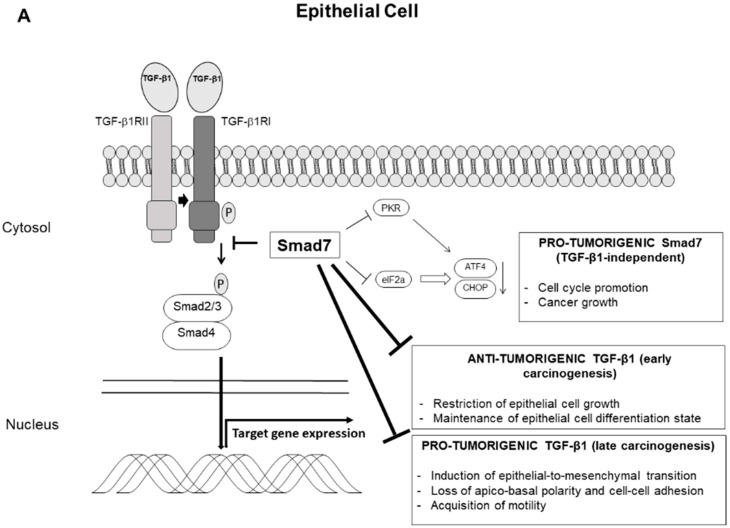

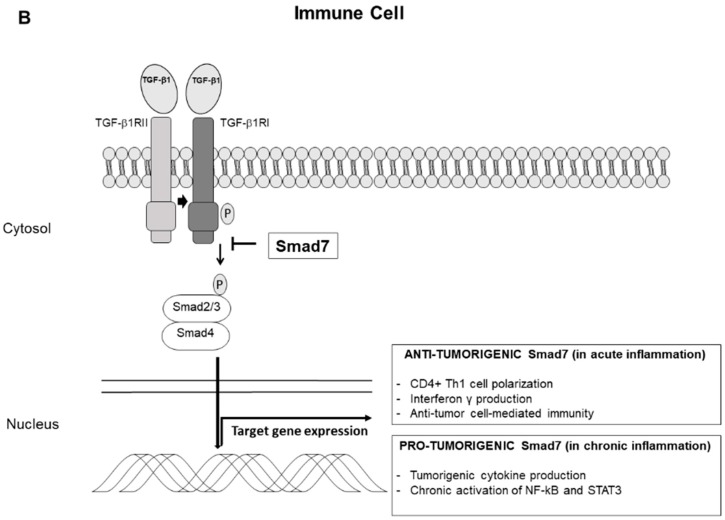
Graphic representation of the Transforming Growth Factor (TGF)-β1/Smad7 signaling and the contrasting effects in epithelial cells (**A**) and immune cells (**B**) on carcinogenesis. Smad7 binds to TGF-β receptor type I and prevents TGF-β1-driven Smad2/3 phosphorylation (P), impairing the transcription of the TGF-β1 target genes and opposing the pro-tumorigenic or anti-tumorigenic effects of TGF-β1, depending on the stage of carcinogenesis. High Smad7 prevents also eukaryotic translation initiation factor-2α (eIF2α) phosphorylation, either directly or through the inhibition of protein kinase RNA (PKR), thus leading to downregulation of transcription factor 4 (ATF4) and CCAAT/enhancer binding protein homology protein (CHOP) with the down-stream effect of stimulating cell cycle progression and cancer cell growth in a TGF-β1-independent manner.

The effects of TGF-β1/Smad7 axis on carcinogenesis are summarized in Figure 1.

## 5. Conclusions

The recent years have witnessed enormous success in CRC pathogenesis and therapy. However, several CRC-related issues remain to be solved and the advanced disease continues to be marked by high rate of mortality. Dissection of the mechanisms by which neoplastic cells grow and metastasize could help identify targets, which can be modulated for therapeutic purposes. One such molecule could be Smad7 given that this protein is over-expressed in sporadic CRC cells and its inhibition associates with reduced cell proliferation and survival. Some important issues, however, need further investigation. For instance, it is still unclear how Smad7 exactly regulates CRC cell growth and death and if the regulatory effect of Smad7 can be influenced by the histologic type and stage of the neoplasia. Further in vivo studies with Smad7 inhibitors would be needed to optimize the delivery of the drug directly to the neoplastic area and establish the biological consequences of Smad7 suppression on the normal, unaffected intestinal epithelium. As pointed-out above, Smad7 is also over-expressed in IBD and CAC, and studies with Smad7-over-expressing mice would seem to suggest an anti-neoplastic effect of Smad7 on this type of neoplasia, as these animals develop more colitis but are resistant to experimental CAC than wild-type mice. Therefore, the blockade of Smad7, at least in theory, could favor rather than inhibit CAC. However, in this context, it is noteworthy that Smad7 is pro-inflammatory in the gut and its suppression attenuates the ongoing mucosal inflammation, which is the major driver for the initiation of CAC. If this comes to be true, we can speculate that Smad7 knockdown could notonly reduce colitis but also limit the occurrence of CAC.
